# Supplementary figures and images for: Antibody responses to Japanese encephalitis virus and dengue virus serotype 2 in children from an orthoflavivirus endemic region after IMOJEV vaccination
Source: PLoS Negl Trop Dis. 2025 Sep 22;19(9):e0013550. doi: 10.1371/journal.pntd.0013550 (PMC12463327; doi:10.1371/journal.pntd.0013550)

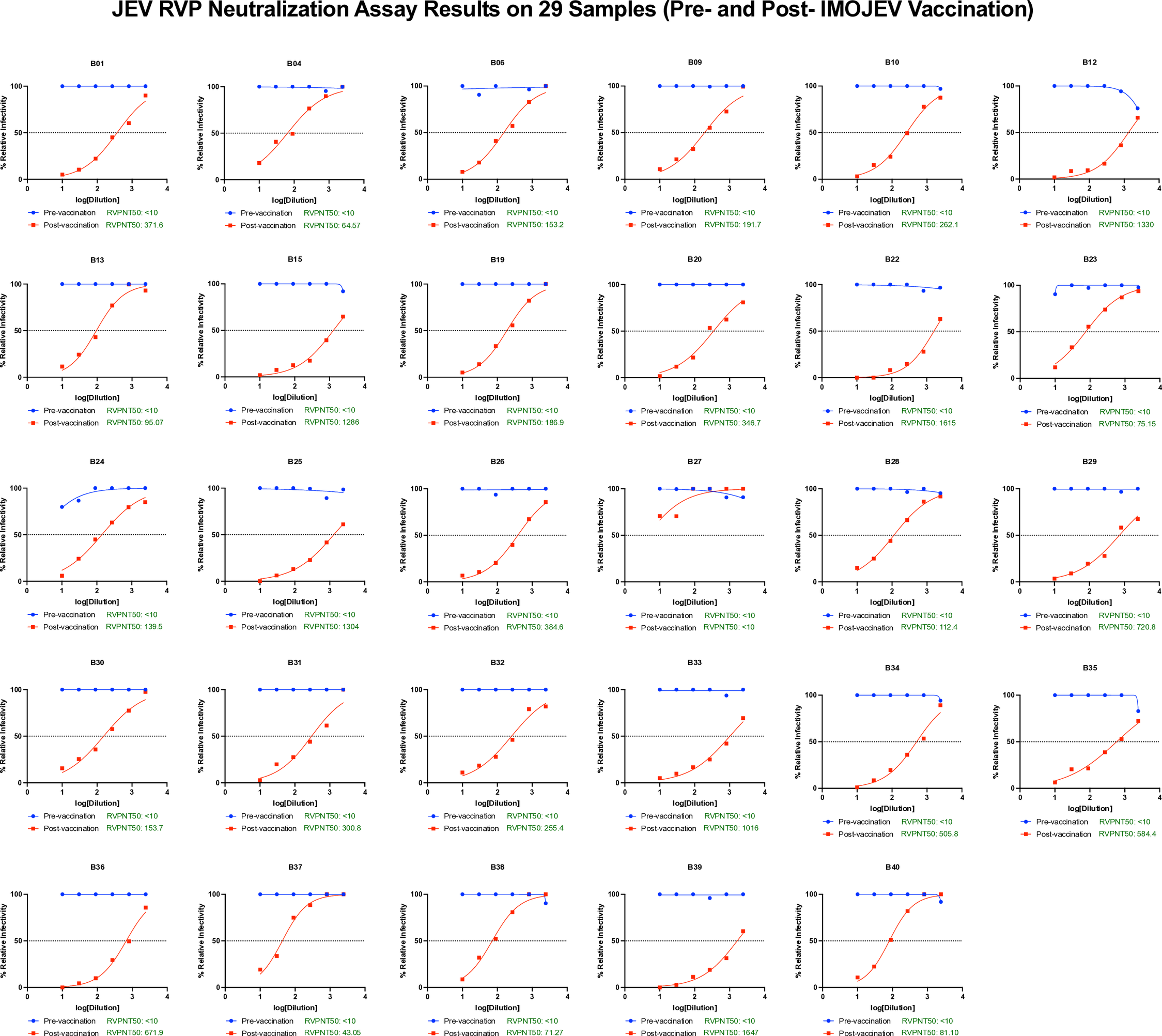

Supplement: S1 Fig — (TIF) [file pntd.0013550.s001.tif]

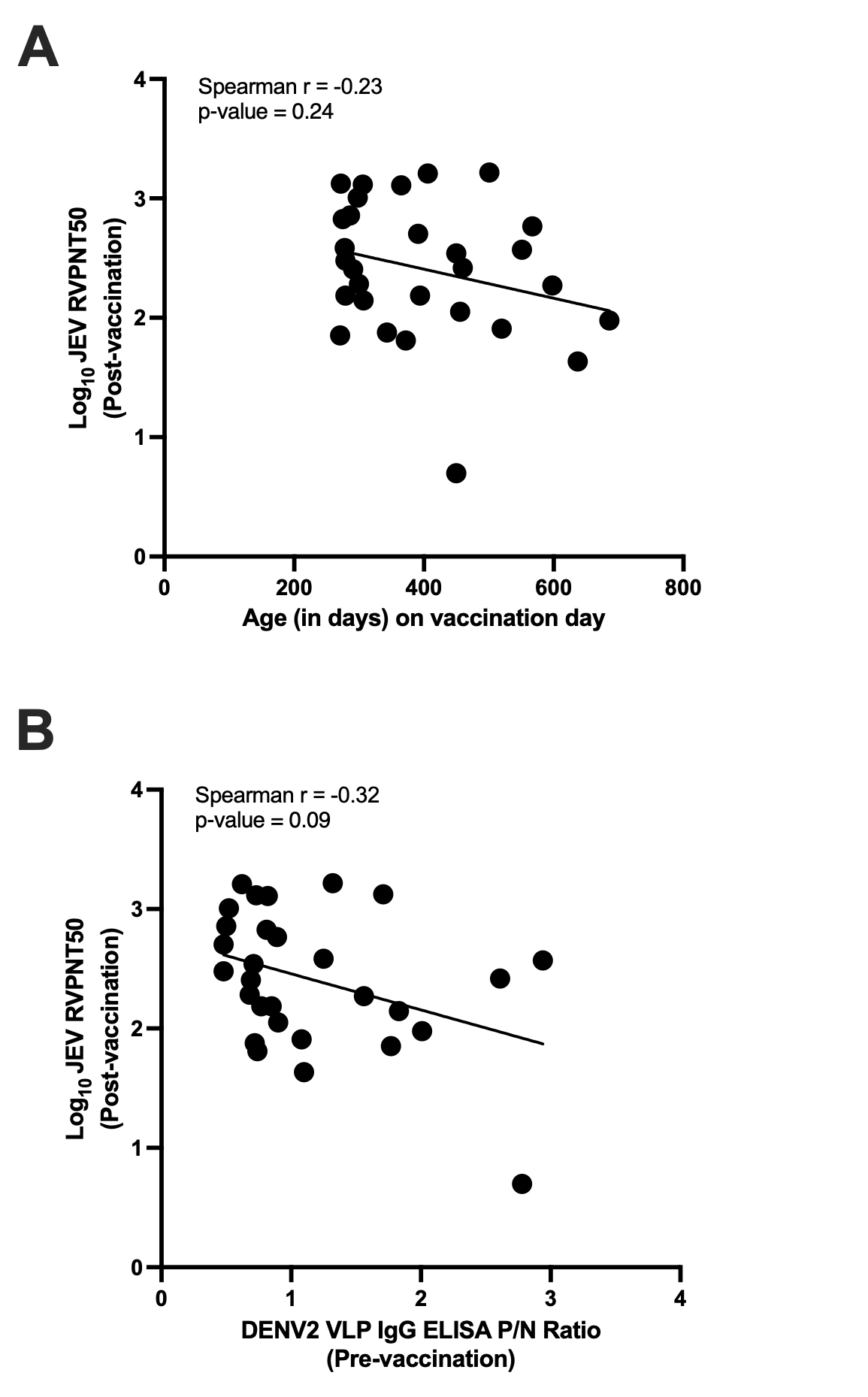

Supplement: S2 Fig — (TIF) [file pntd.0013550.s002.tif]

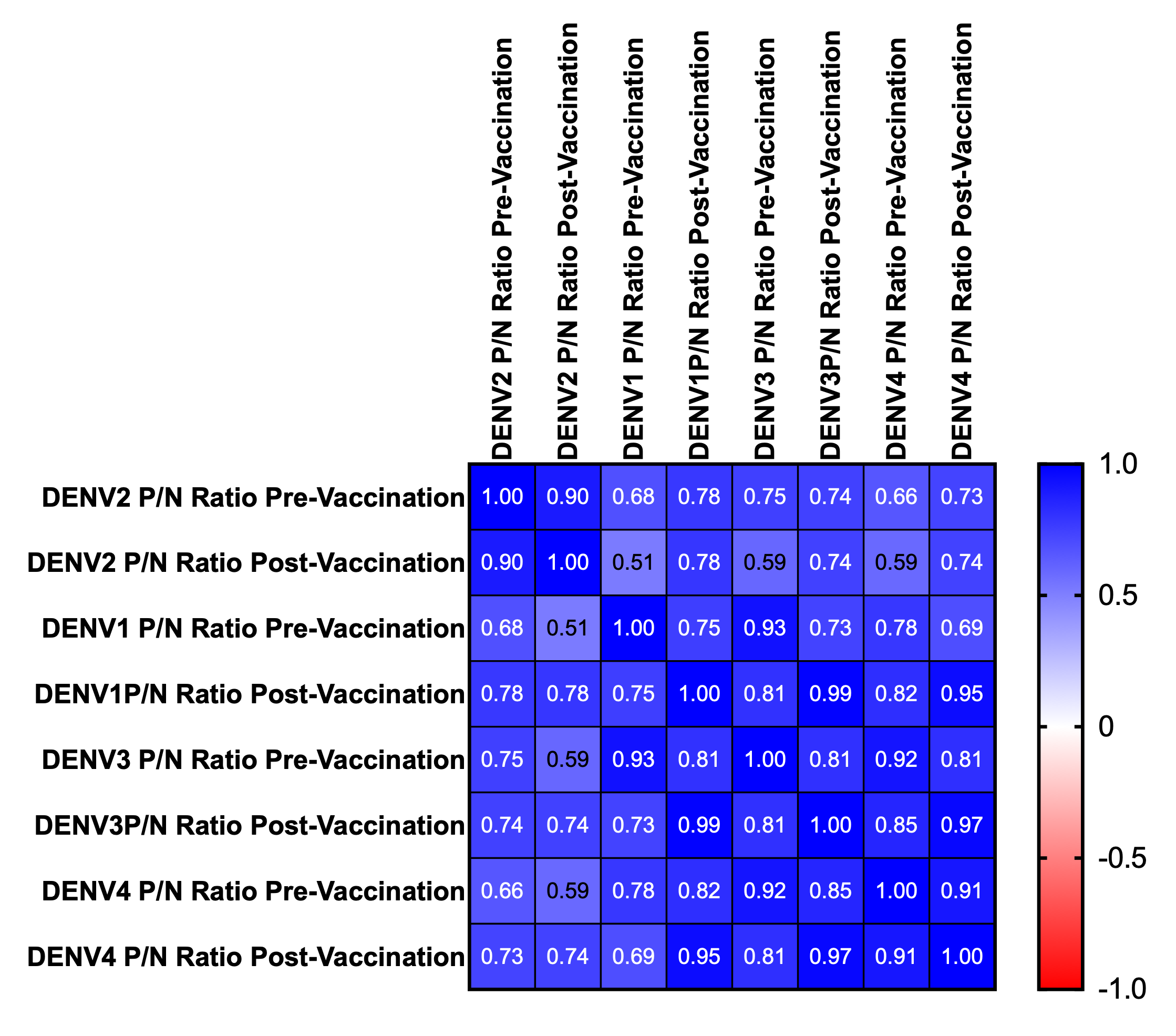

Supplement: S3 Fig — (TIF) [file pntd.0013550.s003.tif]

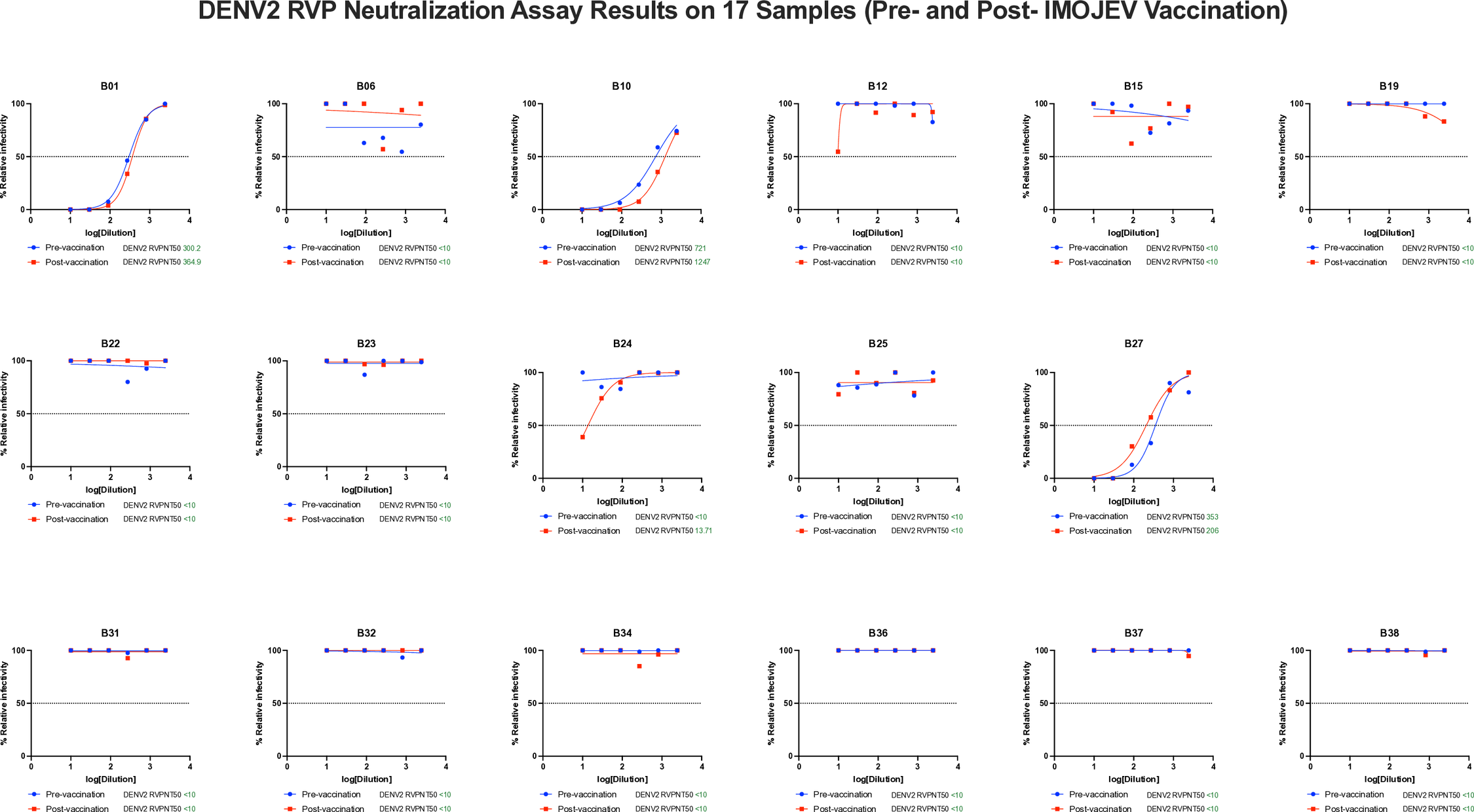

Supplement: S4 Fig — (TIF) [file pntd.0013550.s004.tif]

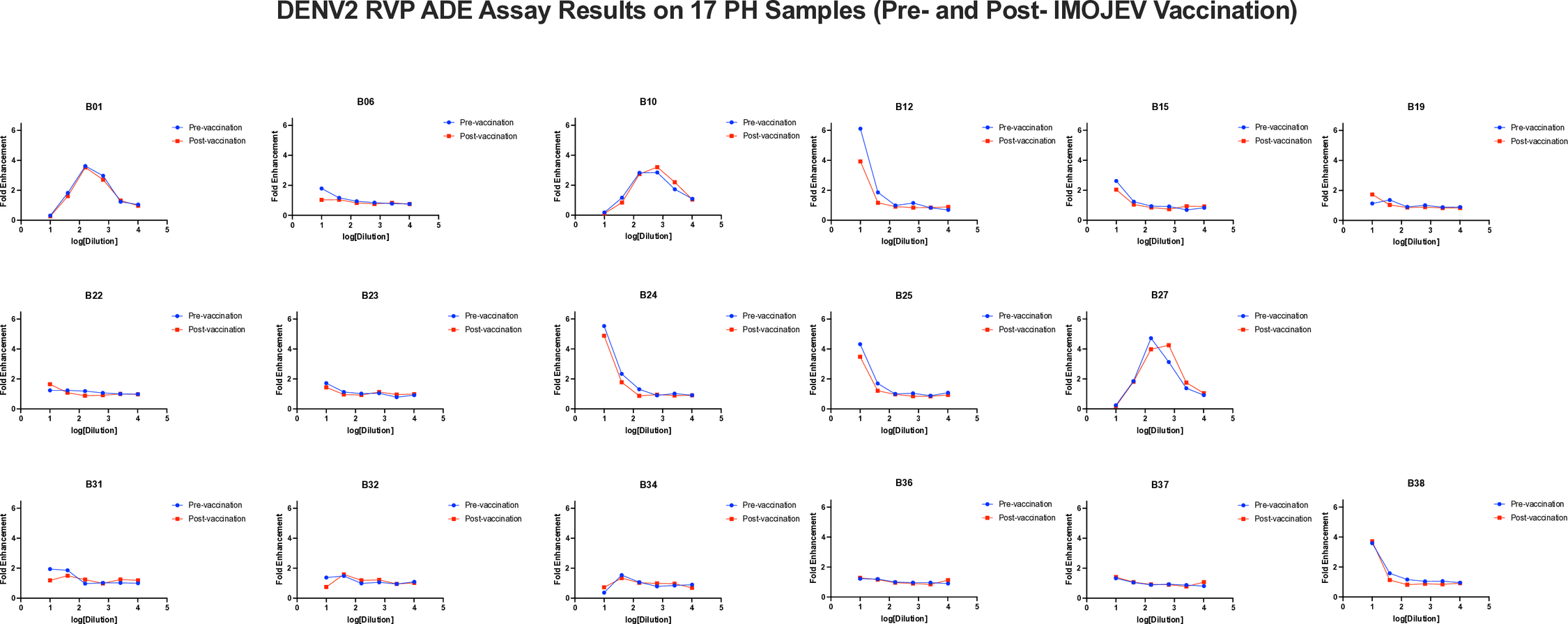

Supplement: S5 Fig — (TIF) [file pntd.0013550.s005.tif]

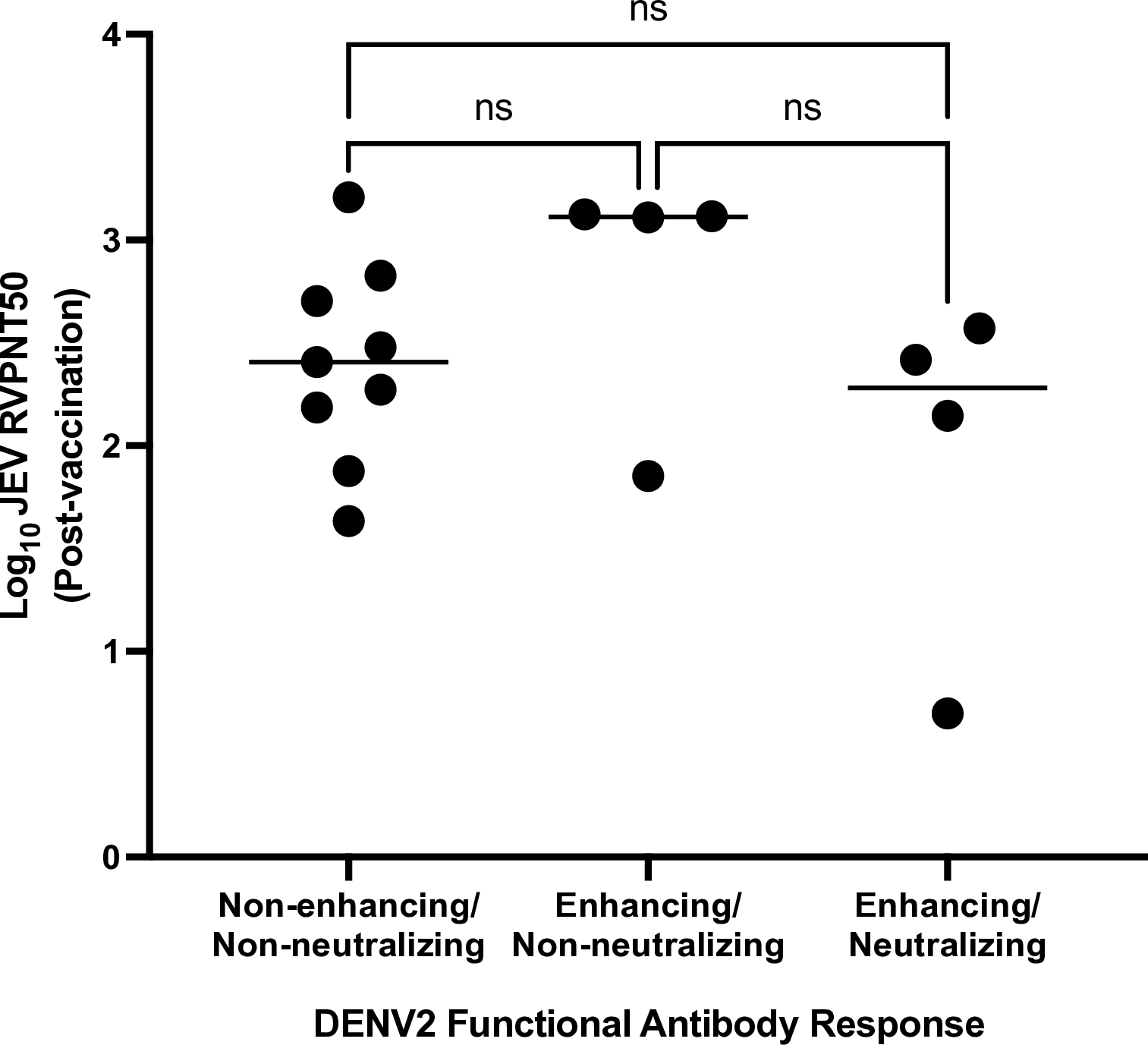

Supplement: S6 Fig — (TIF) [file pntd.0013550.s006.tif]

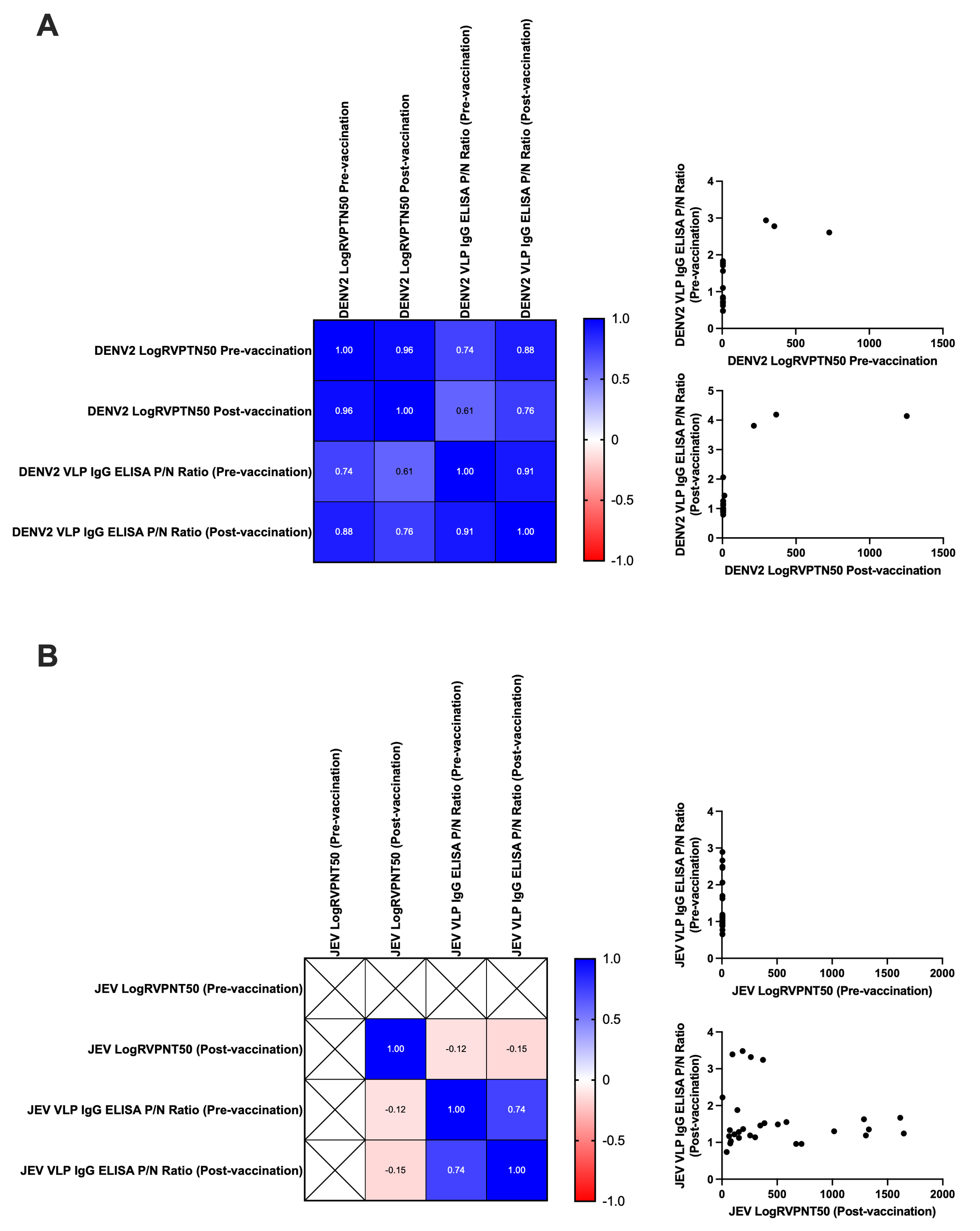

Supplement: S7 Fig — (TIF) [file pntd.0013550.s007.tif]
