# Supplementary material for: Antibody responses to Japanese encephalitis virus and dengue virus serotype 2 in children from an orthoflavivirus endemic region after IMOJEV vaccination
Source: PLoS Negl Trop Dis. 2025 Sep 22;19(9):e0013550. doi: 10.1371/journal.pntd.0013550 (PMC12463327; doi:10.1371/journal.pntd.0013550)
Supplement: Attachment — Submitted filename: Response to Reviewers.docx [file pntd.0013550.s011.docx]

**Editor Comments**

While all reviewer comments should be addressed in full, major methodological issues were raised by two reviewers, particularly in relation to assays used to confirm DENV neutralization. There was also concern regarding the lack of assessment of other DENV serotypes. The methodological issues should be addressed with additional experimental work that provides greater strength to the author’s conclusions.

*We thank the editor and the peer reviewers for their thorough evaluation of this manuscript and for the comments given to further improve this study. We generally agree that readers may be curious about the antibody response to other serotypes and recognize this as a limitation of the current study mainly due to the volume of sera available to us for testing. Because of this, we opted to focus on one serotype with DENV2 chosen due to its prevalence in the Philippines (where all four serotypes circulate) and its relevance to ADE*^1,2^*.*

*We were able to perform additional ELISA assays evaluating antibody reactivity to other DENV serotypes and found that they had a high degree of correlation (Pearson r = 0.66 to 0.78) with the anti-DENV2 antibody binding (see Supplementary Figure 2). While this does not directly answer the questions about functional antibody responses (neutralization and enhancement) to other serotypes, we believe it is sufficient to infer that no significant differences to the DENV2 response would be expected. However, to further address this concern, we modified the mention of “DENV” into “DENV2” throughout the document when pertaining to the results of this study to clarify that we limit our conclusions to this serotype.*

*Please see below our detailed responses.*

**Reviewer #1:**

1. In this study, the authors intended to investigate (1) if the prior DENV infection would affect the JEV vaccination titer and (2) if the JEV vaccination-induced antibody could enhance DENV infection. The authors concluded that “while JEV vaccination increases DENV-binding antibodies, this cross-reactivity does not lead to virus enhancement”. However, the way that the data being presented caused confusion, such as presenting D2VLP-ELISA results as an indication of prior DENV exposure rather than presenting neutralization results. Furthermore, the authors conclude that “while live JEV vaccination increases DENV-binding antibody, this cross-reactivity does not lead to virus enhancement.” The only data to support such a conclusion is the enhancement of DENV-2 RVP without showing the other serotypes of DENV. The overall sample size is also too small to draw significant conclusions.

*Thank you for noting this potential source of confusion. In this paper, we refer to pre-existing DENV antibodies as coming from either (1) natural exposure/infection or (2) maternal transfer. We want to clarify that we wanted to explore the potential effects of* ***both*** *these types of pre-existing antibodies and not those from infection alone, which is why we refer to the ELISA results when talking about binding reactivity in general. We make the difference clear throughout the document and only use “exposure” when referring to the former.*

*-In line 47, we revised the wording to reflect this.*

*-In lines 343-44 and 399-401, the conclusions on “natural DENV exposure” were based on neutralization results.*

*-In line 390, the wording was revised to clarify that orthoflavivirus exposure in the region (i.e., in both children and adults) may be the reason for the observed DENV ELISA results*

*We agree that the small sample size also limits the generalizability of the results and already acknowledged this in the discussion.*

2. The gold standard of defining the prior DENV infection is by performing neutralizing assay. However, throughout the manuscript, including the title, the authors mentioned DENV antibodies, which were actually measured only by DENV-2 RVP. This is a big concern regarding the study design and the conclusion drawn here. First, although the authors provided the D2-VLP-ELISA results, considering its poor sensitivity, how many infants having DENV exposure pre-vaccination need to be determined using all four serotypes of DENV (RVP preferred to be consistent).

*As explained above, we wish to clarify that we used the ELISA results to refer to pre-existing dengue* ***binding*** *antibodies (i.e. from both infection and maternal transfer).*

Secondly, it would make more sense if figure 2E and 2F presented as DENV neutralization positive or negative using all four serotypes of DENV as the indication of prior DENV infection.

*We opted to retain the data as additional ELISA assays against the other dengue serotypes were performed, showing good correlation in terms of binding reactivity values (Supplementary Figure 2). Further, we have revised Figure 2E’s x-axis title and manuscript to reflect that we limit our conclusions to DENV2.*

Third, in figure 3C-3F, it looks like the same groups of infants with pre-vaccinated DENV2 binding antibody have the neutralizing activity against DENV2 RVP and showed enhancement DENV-2 pre and post vaccination (n=8). Therefore, no statistical significance of D2 enhancement is quite clear. However, no results were shown for other serotypes of DENV.

*In line with our responses above, we have limited our study conclusions to DENV2.*

Fourth, since these infants also respond to JEV vaccination with good JEV antibody titers (except B27), the polyclonal sera include both DENV (not limited to DENV2) and JEV antibodies, performing antigen-specific IgG depletion assay by using DENV or JEV from pre- or post-vaccination sera would answer the question if JEV vaccine-induced antibody could enhance the DENV infection and vice versa.

*Thank you for this comment. Unfortunately, such an experiment while ideal, is not possible to perform in our setting due to both resource and sample constraints. However, we think that the presented results, with the addition of the ELISA for other dengue serotypes, sufficiently support our conclusion.*

3. Line 253-254, the authors concluded that the lack of sensitivity of JEV-VLP-based ELISA in detecting vaccine-induced antibodies. No reference has been cited or any preliminary data was performed to determine the validity of the assay, such as how much antigens are needed for coating, why 10 or 25 ng/well for JEV or D2. I wonder if the authors tried to use commercial kit for validation.

*The in-house DENV VLP ELISA was already previously established in the laboratory*^3^*, which served as the basis for the coating antigen concentrations used this study.*

*For JEV, a higher concentration was used due to the reports of poor assay sensitivity in detecting JEV IgG antibodies from vaccination*^4–6^*. There were already cited in line 394. Notably, these studies used commercial kits, which is why they were not used for validation in this study. We instead opted for an in house ELISA, similar to other groups*^7,8^*. However, these studies used JEV NS1 for the detection of natural infection. This cannot be used for our cohort because the IMOJEV vaccine uses the YFV non-structural proteins as its backbone.*

*As such, we used JEV VLP with an OD of at least 1.0 in the positive control as a benchmark for adequate antigen concentration as done by others*^9^*. Our positive control shows an OD > 1.0 while the negative control is at ~0.2, which we think shows reasonable proof of the validity of our assay.*

*We also present in Supplementary Table 2 the results of a preliminary assay where varying JEV antigen concentrations (10 ng/25 ng/50 ng) were used, but the assay still failed to detect the JEV vaccine response.*

4. In this study, the authors used RVP with the backbone from WNV but the structure protein gene derived from either DENV-2 (strain 16681) or JEV (Strain 7812474). Since strain 16681 is an old strain and no longer circulates, the authors should test the enhancement of current circulation strains in the Philippines to reflect the concern of vaccine-induced severe diseases. Similarly, please provide further information if strain 7812474 is similar to the JEV circulating in the Philippines and if it belongs to the same genotype of vaccine strain SA14-14-2.

*The rationale for using DENV 2 strain 16681 is because it is a well-characterized prototype strain. Further, it is the same strain used in the VLP ELISA. JEV strain 7812474 and JEV strain SA-14-14-2 both belong to Genotype III.*

5. Did the authors acquire information about whether the recruited subjects received Dengvaxia or YFV 17D vaccination before? Such information should be revealed in the manuscript.

*Yes, flavivirus vaccination history was collected. We agree with this comment and have included the mention of this important exclusion criterion in the methodology.*

6. The statistical tests require further consultation with the statistician. For example, why use a one-tailed student t-test or Spearman test instead of a two-tailed one? On several occasions, when the sample size is small, the Fisher exact test should be explored.

*Thank you for the comment. We re-evaluated our statistical analyses and made the following revisions:*

- *For Figure 1, all analyses were changed to two-tailed tests. This only changed the statistical significance for Figure 1B (also reflected in Lines 399-401) with no effect on the study conclusions.*
- *For Figure 2, one-tailed t-tests were maintained for comparisons between pre- and post-vaccination sera since here, we are testing a directional hypothesis on whether vaccination increases the anti-JEV antibodies (Figures 2A and 2B). All others were changed to two-tailed tests. There is a minor revision for the discussion about Figure 2F, (Lines 45-46, 142-143 and 418-421), but this does not affect the study conclusions as the correlation was already initially recognized to be weak.*
- *For Figure 3, one-tailed t-tests were maintained for most since we hypothesized that anti-dengue antibodies would increase post-vaccination (Figures 3A and 3B) and that DENV2 neutralizing/enhancing children would have higher DENV2 binding reactivity than DENV2 non-neutralizing/non-enhancing children (Figures 3C and 3E). Figures 3D and 3F were revised to two-tailed tests, but this did not affect the study conclusions.*

*We would also like to acknowledge that upon review, Figures 1A, 1C, 2C were mislabeled as one-tailed in the initial submission. Thus, p-values for these figures were no longer changed in the text.*

*We respectfully disagree regarding the suggestion to use the Fisher’s exact test since no categorical variables were compared in this study.*

7. Could the authors explain why only 17 subjects were selected for post vaccination DENV antibody reactivity experiments since the total 29 subjects were not too big?

*This was largely due to a limitation in the volume of serum samples available to us for testing.*

8. The discussion on Line 360-367 tends to over-explain the data.

*Thank you for the suggestion. We have revised the discussion to make it more concise and focused while retaining key hypotheses we arrived at based on the data.*

9. Throughout the manuscript, flaviruses should be corrected as orthoflaviviruses based on the recent ICTV classification.

*We agree with the use of the updated nomenclature and updated the terminology used throughout the document.*

10. Line 70-71, not just DENV and JEV are prevalent in tropical countries. ZIKV as well and should be included in the sentence.

*We agree that ZIKV is another prevalent flavivirus in the region and have added this statement as well as a reference to support it to the manuscript.*

11. Line 86, DENV is “carried” should be corrected as “transmitted”

*Thank you for the correction. This is now reflected in the manuscript.*

**Reviewer #2:**

The manuscript written by Vista et al. describes about analyzing antibody response patterns against DENV and JEV after immunization with attenuated-chimeric JEV vaccine. The serum samples were collected from Filipino children and were subjected to ELISA, neutralization test and ADE assay. Their results suggested that IMOJEV did not induce ADE against DENV-2 in DENV-seronegative children and did not affect the pattern of dose-response enhancing activity curves in the seropositive individuals. Authors concluded that IMOJEV were able to induce neutralizing antibody against JEV in Filipino children without induction of ADE against DENV-2. Although the sample size is small as mentioned by authors, the present information may be important for children and their parents who are concerning and doubting whether JEV vaccine might induce ADE or not. Specific comments and suggestions are described below.

1. In this paper, antibody levels were mostly indicated by ELISA ratio and PRNT50 titer, and both data were mixed across figures, which sometimes made it difficult to grasp the whole story. For instance, in Fig. 1C, JEV antibody levels were determined by ELISA for pre-vaccination samples, while in Fig. 2C, JEV antibody levels were determined by PRNT for post-vaccination samples. This way may not bring benefits to readers for comparing data between pre- and post-vaccination samples. Such expression needs to be improved.

*We thank Reviewer #2 for their assessment of this work and their thoughtful comments on this study. We hope to address the confusion over the ELISA and PRNT data by clarifying how we organized the data presentation flow in this paper:*

*In Figure 1, we wanted to show the* ***baseline*** *orthoflavivirus reactivity to DENV and JEV based on ELISA. All results reflected in this figure are for pre-vaccination samples.*

*Meanwhile, we focused on the* ***effect of vaccination on anti-JEV antibodies*** *in Figure 2 and the* ***effect of vaccination on anti-DENV antibodies*** *in Figure 3. Pre- and post-vaccination results for both ELISA and neutralization assays (Figures 2A vs. 2B and Figures 3A vs. 3D) are shown and can be used for comparison.*

2. I did not feel very much the necessity of ELISA data in this paper, since NT activity was demonstrated to be strongly correlated with ADE activity. To persuade the need for ELISA data, please show correlation in antibody level between ELISA ratio and PRNT50 titer when DENV antigen was used (as well JEV antigen was used)

*In our viewpoint, the primary value of the ELISA assays in this study is to support the findings of other groups*^4–6^ *that standard JEV IgG ELISAs may not be a good measure of vaccination response as it tends to reflect DENV cross-reactivity rather than true JEV binding. This can only be concluded by showing both DENV ELISA and JEV ELISA results and by comparing it to the neutralization assays as the gold standard.*

*A supplementary figure (Supplementary Figure 6) with the correlation matrix for the ELISA and PRNT results was added, showing that correlation between the two assays is high for DENV but not for JEV.*

3. Minor comment: Statement about Fig. 3F is missing from the manuscript.

*Thank you for noting this oversight. We have added a reference to Figure 3F in the results section.*

**Reviewer #3:**

1. The methodologies described were sound and appropriately referenced.
   The study is well designed, and all limitations were discussed.
2. The results are well presented, clear and cohesive with the analysis plan
3. The findings are described well without overstating the conclusion.
4. This manuscript is very well written with good study aims and significance.

*We sincerely appreciate Reviewer #3’s encouraging feedback on our study.*

*References*

1.	Guzman MG, Vazquez S. The Complexity of Antibody-Dependent Enhancement of Dengue Virus Infection. *Viruses*. 2010;2(12):2649-2662. doi:10.3390/v2122649

2.	Balingit JC, Dimamay MPS, Suzuki R, et al. Role of pre-existing immunity in driving the dengue virus serotype 2 genotype shift in the Philippines: A retrospective analysis of serological data. *International Journal of Infectious Diseases*. 2024;139:59-68. doi:10.1016/j.ijid.2023.11.025

3.	Sanchez-Vargas LA, Mathew A, Salje H, et al. Protective Role of NS1-Specific Antibodies in the Immune Response to Dengue Virus Through Antibody-Dependent Cellular Cytotoxicity. *The Journal of Infectious Diseases*. 2024;230(5):1147-1156. doi:10.1093/infdis/jiae137

4.	Kinsella P, Moso M, Martin G, et al. Laboratory evaluation of ELISA and indirect immunofluorescence assay in response to emergence of Japanese encephalitis virus genotype IV in Australia. *Journal of Clinical Virology*. 2023;168:105580. doi:10.1016/j.jcv.2023.105580

5.	Litzba N, Klade CS, Lederer S, Niedrig M. Evaluation of Serological Diagnostic Test Systems Assessing the Immune Response to Japanese Encephalitis Vaccination. *PLOS Neglected Tropical Diseases*. 2010;4(11):e883. doi:10.1371/journal.pntd.0000883

6.	Cha GW, Cho JE, Ju YR, et al. Comparison of Four Serological Tests for Detecting Antibodies to Japanese Encephalitis Virus after Vaccination in Children. *Osong Public Health and Research Perspectives*. 2014;5(5):286-291. doi:10.1016/j.phrp.2014.08.003

7.	Konishi E, Kitai Y. Detection by ELISA of antibodies to Japanese encephalitis virus nonstructural 1 protein induced in subclinically infected humans. *Vaccine*. 2009;27(50):7053-7058. doi:10.1016/j.vaccine.2009.09.064

8.	Konishi E, Kitai Y, Tabei Y, Nishimura K, Harada S. Natural Japanese encephalitis virus infection among humans in west and east Japan shows the need to continue a vaccination program. *Vaccine*. 2010;28(14):2664-2670. doi:10.1016/j.vaccine.2010.01.008

9.	Galula JU, Salem GM, Destura RV, Remenyi R, Chao DY. Comparable Accuracies of Nonstructural Protein 1- and Envelope Protein-Based Enzyme-Linked Immunosorbent Assays in Detecting Anti-Dengue Immunoglobulin G Antibodies. *Diagnostics*. 2021;11(5):741. doi:10.3390/diagnostics11050741

10. Information on Dengvaxia. Accessed April 4, 2025. https://www.sanofi.com/en/our-company/sustainability/responsible-business-values/information-on-dengvaxia
