# Supplementary material for: Antibody responses to Japanese encephalitis virus and dengue virus serotype 2 in children from an orthoflavivirus endemic region after IMOJEV vaccination
Source: PLoS Negl Trop Dis. 2025 Sep 22;19(9):e0013550. doi: 10.1371/journal.pntd.0013550 (PMC12463327; doi:10.1371/journal.pntd.0013550)
Supplement: Attachment — Submitted filename: 2025_08_011Response to Reviewers+ALR.docx [file pntd.0013550.s012.docx]

**Editor Comments**

Please ensure that all Reviewer 4 comments are addressed.

*We are grateful for the additional feedback for the improvement of this manuscript. Please see our detailed responses below.*

**Reviewer #4:**

This is an interesting study to assess the Ab responses to a live JEV vaccine administered to young Philippino children (9-24 months of age) living in an area with endemic circulation of DENVs, JEV, Zika. The investigators conducted the study to support broader use of JEV vaccines in the country and to address concerns about a JEV vaccine enhancing dengue.

The main questions they address consist of

1) Does pre-existing flavivirus immunity suppress or enhance the JEV vaccine response?
2) Does the JEV vaccine stimulate cross reactive binding and functional Abs to dengue type 2?

3) Does the JEV vaccine stimulate antibodies with the potential to enhance DV2 infections

The investigators obtain clear answers to the first 2 questions. They observe that prior flavivirus immunity has no impact on vaccine take or magnitude of the JEV NAb response. They also observe no boosting of DV2 NAbs by the JEV vaccine.
The studies to answer the 3rd question are misguided and poorly interpreted. Cell culture ADE assays have no predictive value for immune enhanced dengue disease. See my comments below.

Finally, while this is a straightforward study, the results are poorly presented, and the reader is left quite confused about the results and their implications. See my comments below.

Specific comments:

1) The investigators appear to be unfamiliar with basic aspects of flavivirus serology, and the study is not presented in a proper context. The investigators determine dengue immune status before vaccination by testing for DV2 binding Abs. They observe 8/28 DV2 positive children. For the entire paper they consider these children to be DV2 immune. If they are consistent, they would also call these children JEV immune because 7/8 are also positive for JEV binding Abs. The VLP binding assays do not have the required specificity for determining virus-specific immunity because of extensive cross reactivity. Please use the term flavivirus immune because the binding Abs could be the result of a past exposure to any DV serotype, JEV or Zika. The assay for determining virus specific immunity is the neutralization assay. Only 3/8 children with binding Abs actually neutralized DV2. Please revise the paper to reflect the fact that 8/28 kids were flavi immune at baseline and only 3 of these kids were actually DV2 immune.

*We thank the reviewer for their detailed feedback on this manuscript and for recognizing the significance of the questions being addressed. We agree that virus-specific immunity is best measured using the neutralization assay and that the VLP ELISA results may reflect flavivirus cross-reactivity. We believe our paper already sufficiently reflected these points and wish to make the following clarifications:*

(1) *There was one mention of “pre-existing DENV2 immunity” in the Introduction, which has been revised. Elsewhere in the manuscript, children who were deemed DENV+ based on the DENV2 VLP ELISA* ***were not*** *referred to as DENV-immune. Rather, we referred to them as DENV-****reactive****, recognizing that binding reactivity and functional antibody responses such as neutralization/enhancement are distinct. This is also why we respectfully decline to the use of the term “flavivirus immune” to describe ELISA reactive children since immunity would imply protection.*
(2) *The issue of cross-reactivity was already addressed in Figure 1E and in subsequent discussions referring to it (see Lines 341-344).*

2) The finding that nearly all the children respond to the vaccine and develop JEV NAb, with minimal impact on DV2 NAb is important but lost in the way the results are presented. Please consider reorganizing the figures by presenting results relevant to specific questions? In the current paper, many graphs with different comparisons are presented in an indiscriminate manner.

*In our previous response to the reviewers, we clarified the logic behind the current figure arrangements as follows:*

*“In Figure 1, we wanted to show the* ***baseline*** *orthoflavivirus reactivity to DENV and JEV based on ELISA. All results reflected in this figure are for pre-vaccination samples.*

*Meanwhile, we focused on the* ***effect of vaccination on anti-JEV antibodies*** *in Figure 2 and the* ***effect of vaccination on anti-DENV antibodies*** *in Figure 3.”*

*We considered the reviewer’s comment on re-arranging the figures according to the three specific questions but since only a subset of children were included in the neutralization/ADE assays (due to sample volume limitations), we found that it is best to present ELISA results first before the functional assays. We also moved the Spearman correlations to Supplementary Figure 2 and removed the ELISA comparison between groups in Figure 3.*

*Revisions were made in the Abstract (Lines 41-44), Introduction (Lines 121-124), and Discussion (Lines 334-336) to clarify the major findings as suggested.*

3) The JEV vaccine drives a NAb response in nearly all children, but this is not reflected in the JEV VLP binding assay, where many children remain negative. This could be due to vaccine induced IgM competing with IgG or a technical issue with the assay. Please discuss.

*We agree with the reviewer that this is another possible explanation as the literature also shows that at one-month post-vaccination, anti-JEV IgM may persist in around half of vaccinees. This was added to the discussion (Lines 349-351).*

4) As already mentioned, cell culture ADE is not predictive of risk in people. Even the cell culture ADE measurements are confusing. The investigators choose fold enhancement. Why do some sera enhance at the highest concentration tested while others require significant dilution of the serum. If one serum sample enhanced 5 fold at a dilution of 1:20 only and another serum reaches a peak of 5 fold enhancement at a serum dilution of 1:200, as presente din this paper they both have a peak fold enhancement of 5. Are they really the same? If the authors look closely at the ADE curves, they will see that kids with high DV2 neut titers have to be diluted to enhance while those with no NAb sometimes enhance at the highest concertation tested. What does this mean with respect to JEV vaccination enhancing DV2 in vivo? The only message I take from these data are that the cell culture ADE profiles remain the same before and after vaccination suggesting they are being driven by immunity that existed before vaccination and not Abs stimulated by the vaccine.

*Supplementary Figure 4 supports the reviewer’s comment that ADE curves “remain the same” pre- and post-vaccination.*

*In the literature, ADE profiles tend to be represented differently across the literature with some using fold enhancement ^1,2^ while others use an “ADE titer”, defined as “the maximum serum dilution factor exceeding the ADE threshold”^3^.*

*In our study, comparing the pre- and post- peak fold enhancement (Figure 3D) as well as the serum dilution at peak fold enhancement conveys the same take home message (that DENV2 enhancement is unaffected by JEV vaccination). Additional data (Table 1) was provided to further support this.*

I hope these comments are useful to reorganize and rewrite this paper to highlight the important results and conclusions of this study.

*The comments are highly appreciated, and we hope the reviewer finds these responses satisfactory.*

References

1.	Saito Y, Moi ML, Takeshita N, et al. Japanese encephalitis vaccine-facilitated dengue virus infection-enhancement antibody in adults. *BMC Infect Dis*. 2016;16(1):578. doi:10.1186/s12879-016-1873-8

2.	Putvatana R, Yoksan S, Chayayodhin T, Bhamarapravati N, Halstead SB. Absence of Dengue 2 Infection Enhancement in Human Sera Containing Japanese Encephalitis Antibodies. *The American Journal of Tropical Medicine and Hygiene*. 1984;33(2):288-294. doi:10.4269/ajtmh.1984.33.288

3.	Kotaki T, Nagai Y, Yamanaka A, Konishi E, Kameoka M. Japanese Encephalitis DNA Vaccines with Epitope Modification Reduce the Induction of Cross-Reactive Antibodies against Dengue Virus and Antibody-Dependent Enhancement of Dengue Virus Infection. *Vaccines*. 2022;10(9):1411. doi:10.3390/vaccines10091411
